# Randomly stopped sums: models and psychological applications

**DOI:** 10.3389/fpsyg.2014.01279

**Published:** 2014-11-10

**Authors:** Michael Smithson, Yiyun Shou

**Affiliations:** Research School of Psychology, The Australian National UniversityCanberra, ACT, Australia

**Keywords:** random sums, response time modeling, financial loss modeling, decision modeling, accumulation models of decision-making

## Abstract

This paper describes an approach to modeling the sums of a continuous random variable over a number of measurement occasions when the number of occasions also is a random variable. A typical example is summing the amounts of time spent attending to pieces of information in an information search task leading to a decision to obtain the total time taken to decide. Although there is a large literature on randomly stopped sums in financial statistics, it is largely absent from psychology. The paper begins with the standard modeling approaches used in financial statistics, and then extends them in two ways. First, the randomly stopped sums are modeled as “life distributions” such as the gamma or log-normal distribution. A simulation study investigates Type I error rate accuracy and power for gamma and log-normal versions of this model. Second, a Bayesian hierarchical approach is used for constructing an appropriate general linear model of the sums. Model diagnostics are discussed, and three illustrations are presented from real datasets.

This paper presents an approach to modeling the sums of a continuous random variable over a number of measurement occasions when the number of occasions also is a random variable, based on models of this kind used in financial statistics. These sums are known as “randomly stopped sums.” Typical examples of randomly stopped sums in psychology are summing the durations of fixations in an eye-tracking study for each subject to obtain the total amount of time spent attending to a stimulus, summing the amounts of time spent attending to pieces of information in an information search task leading to a decision to obtain the total time taken to decide, or summing the amounts of money spent per month on a particular type of consumer item. Although there is a large literature on this kind of variable in financial statistics, it is almost completely absent from psychology. Our treatment departs from standard methods for modeling sums of magnitudes (usually losses or gains) in financial portfolios in two respects. First, it emphasizes combining effects from the frequency and magnitude models in the models for the sums. Second, it incorporates a Bayesian hierarchical approach to constructing an appropriate general linear model for the conditional distribution of the sums.

We begin by presenting an example of this model, after which we develop the model framework and briefly review the methods used in financial statistics for parameter estimation. We then present an example to illustrate the model and also demonstrate its utility. Thereafter, a section on model estimation and diagnosis describes a simulation-based method and a Bayesian MCMC method for parameter estimation, and describes some advantages for the Bayesian approach over the simulation approach. This section also includes a third example, illustrating the Bayesian method. We then report a simulation study investigating Type I error rate accuracy and power for the gamma and log-normal versions of the model. The paper concludes with an example of a three-level model highlighting the advantages of a Bayesian approach to hierarchical models.

## 1. Model framework

### 1.1. Motivating example

To our knowledge, the paper by Aribarg et al. (2010) presents the only example of a randomly stopped sums model in the psychological literature. Their model is a special case of the models elaborated in this paper, so we will begin by describing their model and its application. They investigated the relationship between consumers' attention to print advertisements and subsequent advertisement recognition measures, using eye-tracking to measure attention. Their study had *I* = 185 subjects, *J* = 3 advertisement design elements (pictorial, text, and brand), *L* = 48 advertisements. The relevant eye-tracker data consisted of the number of fixations and individual fixation durations for participant *i* on element *j* of advertisement *l*.

Aribarg et al. proposed an attention model of gaze duration, *S_ijl_*, “as the sum of individual fixation durations through a hierarchical randomly stopped sum Poisson model” (Aribarg et al., 2010: 390). They assumed a Poisson distribution for the marginal distribution of fixation frequency *n_ijl_*, with a parameter λ_ijl_; and they assumed an exponential distribution for the fixation durations *Z_k_i__jl*, where *k_i_* indexes the individual fixations for the *i*th participant, with a parameter μ_*ijl*_. Conditional on the *n_ijl_*, gaze duration *S_ijl_* is the sum of independent identically distributed (i.i.d.) exponential random variables *Z_k_i__jl*. Therefore, the conditional distribution of *S_ijl_* is a gamma distribution (Johnson et al., 1995) with parameters *n_ijl_* and μ_*ijl*_, and expectation *n_ijl_*μ_*ijl*_.

Finally, Aribarg et al. parameterized λ_ijl_ and μ_*ijl*_ as functions of explanatory variables with random intercepts and coefficients, using the log link function for both parameters:

$$\begin{array}{l} \;\log\left(\lambda_{ijl}\right)=\sum_{m}\alpha_{ij}x_{ijlm}\\ \log\left(\mu_{ijl}\right)=\gamma_{ij} \end{array}$$

where the *x_ijl_* are the explanatory variables for the expected fixation frequency. The fixation durations were modeled only with random intercepts.

Although Aribarg et al. arrived at the conditional distribution of gaze duration *S_ijl_*, they did not describe the marginal distribution which, as we shall see, involves an infinite sum. Nor did their model include predictors of the fixation durations. We therefore turn now to elaborating and generalizing the randomly stopped sums model.

### 1.2. General model

For simplicity but without loss of generality, we consider just a two-level data structure with *J* subjects, each of which has *n_j_* i.i.d. continuous random variables *Z_ij_*, where *n_j_* is a realization of an integer-valued random variable, *N_j_*. The sum of *N_j_* i.i.d. continuous random variables *Z_ij_* for subject *j* is determined by the distribution of *Z_ij_* magnitudes for *j* = 1, …, *N_j_* and the frequency distribution of *N_j_*. Denoting the sum by *S_j_* = *Z*_1*j*_ + … + *Z_N_j_j_*, the sums cumulative distribution function (cdf) is

(1) $$F_{s}\left(s_{j}\right)=\mathrm{Pr}\left(S_{j}<s_{j}\right)=\sum_{n=0}^{\infty }\mathrm{Pr}\left(S_{j}<s_{j}|N_{j}=n_{j}\right)\pi_{nj},$$

where π_*nj*_ = Pr(*N_j_* = *n_j_*). In turn, Pr(*S_j_* < *s_j_*|*N_j_* = *n_j_*) is the n-fold convolution of the cdf of the *Z_ij_*.

Suppose that in the frequency model for the *j*th subject, *N_j_* has a distribution with central tendency *h*^−1^(λ_*j*_) where *h* is an appropriate link function, and the model for λ_*j*_ is

(2) $$\lambda_{j}=\sum_{k}\alpha_{k}x_{kj},$$

where the *x_kj_* are predictors and the α_*k*_ are coefficients. Likewise, suppose that the magnitudes for the *j*th subject, the *Z_ij_*, have a distribution with a central tendency parameter *E*(*Z_ij_*) = *g*^−1^(ν_*ij*_) where *g* is an appropriate link function, and the model for ν_*ij*_ is

(3) $$\nu_{ij}=\mu_{j}+u_{i},$$

with *u_i_* ~ *N*(0, σ_*u*_), and

(4) $$\mu_{j}=\sum_{m}\gamma_{mj}y_{mj}.$$

Here, the *y_mj_* are predictors and the γ_*mj*_ are random coefficients, so that

(5) $$\gamma_{mj}=\beta_{m}+\varepsilon_{j},$$

with ε_*j*_ ~ *N*(0, σ_ε_).

Clearly there is no explicit expression for *F_s_*(*s_j_*) in general, so numerical methods have been developed for approximating it. These include Panjer recursion (Panjer, 1981; Klugman et al., 2004), a Fourier transform method for convolutions, two Gaussian-based approximations (Daykin et al., 1994), and simulation from the distributions (Goulet and Pouliot, 2008). R Development Core Team (2013) has a package (Dutang et al., 2008) that implements the five aforementioned methods. The Panjer, Fourier, and simulation methods are worth considering in principle, whereas the two Gaussian methods are insufficient approximations for our purposes because they are not accurate for small to moderate sample sizes. The Fourier method is suited only for problems with few parameters, and the Panjer recursion method typically takes longer than the simulation method and requires discretizing the magnitudes distribution, so we focus on the simulation approach alone. Later we elaborate a Bayesian hierarchical approach to these models.

The inputs into the simulation method are the frequency and magnitude models described above. The output is an approximation of *F_s_*(*s_j_*), and from this we may extract summary statistics such as the expected value of *S_j_* and its quantiles. Moreover, the same simulation results can be used to estimate predictor effects on *S* itself. Denoting the expected value of *S_j_* by *f*^−1^(μ_*Sj*_), where *f* is an appropriate link function, the simulation can provide bootstrap estimates of the coefficients in a model that includes predictors from both the frequency and magnitudes models:

(6) $$\mu_{Sj}=\sum_{k}\delta_{k}x_{kj}+\sum_{m}\eta_{m}y_{mj}.$$

We address the issue of how these coefficients may be related to the coefficients for the frequency and magnitude models below.

We now turn to the choice of distributions for the frequencies and magnitudes. The Aribarg et al. model had a Poisson distribution for the frequencies, and an exponential distribution for the magnitudes. A well-known problem for the Poisson distribution is over-dispersion (see, e.g., Hilbe, 2011), often due to individual differences among subjects. Three popular alternative frequency distributions are available to deal with over-dispersion: The negative binomial, the compound Poisson-gamma, and compound Poisson-log-normal (Johnson et al., 1993). The latter two distributions assign a gamma and a log-normal distribution, respectively, to the λ_*j*_ parameter of the Poisson distribution.

There are distributions for the magnitudes whose sums also follow known distributions conditional on *N_j_* = *n_j_*. Perhaps the most natural example for many psychological applications is a gamma distribution model for the magnitudes, which yields a conditional gamma distribution for the sums. That is, if the *Z_ij_* are distributed gamma(ρ_*j*_, ς_*ij*_), then the distribution of *S_j_* conditional on *N* = *n_j_* is gamma(ρ*_j_n_j_*, ς_*ij*_). We shall denote this as the GG model. The Aribarg et al. (2010) model is a special case because they model the magnitudes with the exponential distribution. Another example, suitable when long-tailed distributions are expected, is the inverse Gaussian (IG) distribution. If the *Z_ij_* are i.i.d. IG(θ_*j*_, κ_*ij*_), then the distribution of *S_j_* conditional on *N* = *n_j_* is IG(*n_j_*θ_*j*_,*n*^2^_*j*_κ_*ij*_). However, there is no need to restrict ourselves to models of this kind. We shall see, for instance, that a log-normal model for the magnitudes and a log-normal model for approximating the sums (the LN model) can be effective, even though the sum of log-normal random variables does not have a log-normal distribution.

Another convenient property that may be desirable for interpretive purposes is the fact that if the frequency, magnitude, and sums models all employ the log link, then the frequency and magnitude model coefficients may be substituted into equation 6 for the sums model coefficients, i.e., α_*k*_ = δ_*k*_ and β_*k*_ = η_*k*_. Moreover, if the frequency and magnitude models share a predictor, *x_k_*, say, then the sums model's coefficient for this predictor will be ω_*k*_ = α_*k*_ + β_*k*_. We will make use of this property throughout this paper. Note that while the log link is not the canonical link function for the GG or IG-IG models described above (although it can be employed with them), it is the canonical link for the LN model.

### 1.3. Another motivating example

We now present a second application of the randomly stopped sums model, with two purposes in mind. First, we wish to illustrate the use of distributions other than those in the Aribarg et al. paper. Second, and more important, we wish to demonstrate the utility of modeling the magnitude sums, which Aribarg et al. do not explicitly do. After all, there is no a priori guarantee that a predictor's effects on frequency and magnitude will “add up” to a significant effect on the magnitude sums, especially if one or both effects are not significant or if they are in opposing directions. The eye-tracker study to be reanalyzed here is a test-case in point.

Owens et al. (2009) described an eye-tracker study analyzing the eye movement patterns of users viewing a portal web page. In their study, the saliency of one of the portal channel titles was manipulated in two different page locations (left and right in the center row) by modifying the color the text. These two manipulation conditions were compared with a control condition in which the title's text color was identical to the rest of the text. The authors found that eye movements were affected by the salient title only when it was located on the left side of the page. However, they based their analysis on only those subjects who had fixated on all six channels above the page fold, and limited their analyses to the order and number of fixations.

Here, we examine the experimental effect on the frequency of fixations, the mean fixation duration, and the durations sum. We use the data from all 57 participants and, for the sake of simplicity, we ignore the channel (fixation location). We handle over-dispersion in the frequencies via a negative binomial GLM, which yields α_1_ = 0.135 for the difference between the left and control conditions (*p* = 0.14), and α_2_ = 0.020 for the difference between the right and control conditions (*p* = 0.83). Thus, neither condition reaches significance, although the left condition is fairly close.

We construct a two-level LN model predicting mean fixation duration, which yields β_1_ = 0.111 for the difference between the left and control conditions, and β_2_ = 0.098 for the difference between the right and control conditions. Again, neither effect achieves significance, with *t* statistics of 1.4 and 1.2, respectively.

However, do the effects of the left condition on frequency and duration combine to a significant effect on the total gaze time? Both of the effects for the left condition are in the same direction. The coefficient for the log mean duration is 0.111 and the coefficient for the log frequency is 0.135, so their combined effect should yield a coefficient of 0.246 if we model the duration sums using the log link. The simulation method mentioned above (and detailed in the next section) with the fixation duration sums modeled as a log-normal random variable and 10,000 runs yields a mean coefficient for the left condition effect of 0.247, very close to what we should expect. The simulation also produces a 95% confidence interval of [0.048, 0.444] for this coefficient. Thus, the concatenation of a non-significant frequency effect and non-significant mean duration effect nevertheless has resulted in a significant effect on the duration sum. The question of when a predictor's effects on frequency and fixation duration combine to produce an effect on the duration sum will be investigated further at several points in this paper, specifically when we examine statistical power.

## 2. Model estimation and diagnostics

### 2.1. Models for the simulation approach

The usual options for modeling counts in the classical framework are the Poisson and negative binomial distributions, with the latter employed to deal with over-dispersion. In the simulation approach presented here, these conventional alternatives, and the model evaluation and diagnostic methods for them, usually will suffice. We shall see that in a Bayesian hierarchical setup there are other possibilities such as a compound Poisson-gamma model for dealing with multi-level data structures.

Likewise, the usual life distributions can be employed to model the magnitude means. In our first example the exponential distribution was employed, whereas in the second example a gamma distribution was used. We discuss the choice of a distribution for the magnitudes both in this section and later in this paper.

The procedure for simulating the sums distribution is best explained in the setting of our two-level data structure, where there are *J* subjects, each containing a random number of magnitudes.

1. Given a marginal frequency distribution model for the *N_j_*, randomly draw *J* values, *n_j_*, from this distribution.
2. Given a conditional distribution for the magnitudes *Z_ij_*, randomly draw *n_j_* values for the *j*th subject and compute the mean, for *j* = 1, …, *J*.
3. Compute the product of each mean with the corresponding *n_j_* to obtain the *J* sums of the magnitudes.

Repeating these three steps many times builds up a distribution of the vector of simulated sums. In our experience, 10,000–20,000 runs produce stable simulation results.

As indicated earlier, if the frequency and magnitude models share all predictors and also use the same link function, then coefficients for these predictors in modeling the sums may be obtained via an appropriate GLM. The most obvious link function is the log, because it enables Equation (6) to hold. A natural choice for the GLM is the GG model, for reasons given earlier. Another viable choice is the LN model, even though the sum of log-normal variables is not a log-normal random variable. This kind of model tends to be favored in the financial statistics literature, along with versions that substitute longer-tailed distributions for the log-normal. There is a literature on approximating sums of log-normal variates with a log-normal model (Dufresne, 2004) which generally views such approximations favorably. Which model is most appropriate for psychological research probably has to be decided by modelers in specific applications, and we shall compare them with the examples in this paper as well as in a simulation study.

Model diagnostics in the simulation approach include the usual diagnostics for the frequency and magnitudes models, i.e., goodness-of-fit measures, residuals, and associated leverage or influence statistics. For example, the log-likelihood for the exponential model of the eye-tracker fixation durations is −15341.0, whereas for a LN model it is −1679.6, suggesting that the LN model fits the durations better. Indeed, the correlation between the predicted and observed magnitudes is 0.991 for the LN model and 0.907 for the exponential model.

For the sums model, however, diagnostics are limited primarily to informal comparisons of the simulated sums distribution with the observed sums distribution. In the Owen et al. study, a 10,000-run simulation yields a mean sum of 9896 and standard deviation 2961 for the LN model. The empirical sums distribution has a mean 10461 and standard deviation 4185, indicating that the LN model somewhat under-estimates the variance. Turning to quantiles, the 25th, 50th, and 75th percentiles of the LN model are 7647, 9586, and 11806, respectively. The corresponding empirical distribution percentiles are 7715, 9923, and 14386. These results suggest that the LN model is more accurate in the lower quantiles but less accurate in the upper quantiles.

Additional diagnostics may be obtained from the GLMs estimated for each of the simulation runs. The distributions of the coefficients can be examined for evidence of pathologies such as multi-modality, skew, and overly strong influence from outliers. For example, in the eye-tracker results, the means and medians of the coefficients for the GLM sums are nearly identical for both the GG and LN models, indicating the absence of skew (and a more formal assessment of skew confirms this).

### 2.2 Bayesian randomly stopped sums models

The frequentist approaches to estimating randomly stopped sums models rely on approximating the sums distribution via simulation, based on the parameter estimates in the frequency and magnitude models. We then estimate the parameters for a conditional model of the simulated sums distribution. Thus, the sums are only indirectly modeled by this approach.

A Bayesian hierarchical modeling framework provides a more direct approach. The observed sums are modeled by an appropriate GLM, whose parameters are functions of the frequency and magnitude models' parameters, which are estimated simultaneously. As a result, we can not only obtain standard errors and credible or highest-density intervals for the sums model parameters, but predictions of the sums that can be compared with the data. Multi-level data structures are easily dealt with in this framework, as will be demonstrated with a three-level data set in the penultimate section, and a more thorough set of diagnostic and model comparison tools are available.

We may handle over-dispersion in the frequencies by allowing the Poisson distribution parameter to follow a gamma distribution, i.e., λ*j* ~ gamma(*q*, *r_j_*). The *r* parameter, in turn, is a function of hyper-parameters:

(7) $$r_{j}=q/q\eta_{j}.$$

The shape parameter *q* is given an uninformative gamma prior. The predictors presented in Equation (2) now determine η*_j_*:

(8) $$\log(\eta_{j})=\sum_{k}\alpha_{k}x_{kj}.$$

The α_*k*_ coefficients are given uninformative Gaussian priors.

As mentioned earlier, a natural model for the magnitudes has them distributed as gamma(*q_m_*, *r_ij_*), with *r_ij_* = *q_m_*/exp(μ*_j_*). Because this model uses the log link, the μ*_j_* are means in the log-scale, with μ_*j*_ ~ *N*(υ_*j*_, σ^2^_υ_), and the predictors in Equation (4) now predicting the υ*_j_*:

(9) $$\upsilon_{j}=\sum_{m}\beta_{m}y_{mj}.$$

Note that the random term for the γ_*mj*_ coefficients in Equation (4) is absorbed by the distribution model for the υ*_j_*. The β*_m_* coefficients are given uninformative Gaussian priors. Finally, the sums are modeled as gamma (λ*_j_q_m_*, *r_ij_*), with *r_ij_* = λ*_j_q_m_*/exp(μ_*Sj*_). The means may be written as

(10) $$\mu_{\text{S}j}=\mu_{j}+\log\left(\lambda_{j}\right).$$

Another way of viewing this equation is via equation 6, where, as mentioned earlier, if the frequency and magnitude models share a predictor then the sums models coefficient for this predictor will be ω*_k_* = α*_k_* + β*_k_*.

Alternatively, the magnitudes may be modeled as log-normal variates with means μ*_j_* in the log-scale and variance σ^2^. Then, the sums are modeled as log-normal variates having means μ_*Sj*_ in the log-scale and variance σ^2^*_s_*. As mentioned earlier, this model is favored in the financial statistics literature.

Applying this method to the eye-tracker example using MCMC estimation in OpenBUGS (for an overview of the BUGS project see Lunn et al., 2009) and two chains results in models that converge well in all parameters after a 5000 iteration burn-in. We now apply a GG and a LN model to the eye-tracker example. Table 1 compares the simulation and Bayesian LN and GG model results, showing that there is reasonably close agreement between them. The parameter estimates are similar, including the coefficients for the sums models. The only difference between the simulation and Bayesian models is in the standard errors for the frequency model parameters, which is due to the use of a negative binomial distribution for the simulation model and a Poisson distribution for the Bayesian model (The latter handles over-dispersion via the gamma-distributed parameter in the Poisson model). The LN and GG models also are in fairly close agreement on their β and ω coefficients (they have identical frequency models, so have nearly identical α coefficients as expected).

**Table 1:** **Simulation vs. Bayesian models**.

	**Simul. Estim**.	**Model *SE***	**Bayes Estim**.	**Model *SE***
**LNORMAL**				
α_0_	3.666	0.065	3.678	0.033
α_1_	0.135	0.092	0.134	0.046
α_2_	0.020	0.094	0.019	0.048
β_0_	5.293	0.055	5.291	0.060
β_1_	0.111	0.079	0.110	0.087
β_2_	0.098	0.080	0.095	0.088
ω_0_	8.957	0.065	8.969	0.068
ω_1_	0.247	0.099	0.244	0.097
ω_2_	0.117	0.101	0.114	0.099
**GAMMA**				
α_0_	3.666	0.065	3.657	0.022
α_1_	0.135	0.092	0.135	0.029
α_2_	0.020	0.094	0.009	0.030
β_0_	5.403	0.059	5.379	0.092
β_1_	0.140	0.084	0.121	0.133
β_2_	0.116	0.086	0.098	0.092
ω_0_	9.029	0.070	9.036	0.092
ω_1_	0.276	0.099	0.256	0.132
ω_2_	0.132	0.101	0.108	0.133

However, the LN and GG models may yield estimates and standard errors that differ enough to raise questions about hypothesis testing or conclusions. In circumstances such as this, model diagnostics, evaluation, and comparison may help researchers decide among disagreeing models.

### 2.3. Decision study

The third example presented here is a study of deliberative decision making. Tang (2013) investigated the impact of choice set characteristics on the time taken for participants to choose a charity for making a donation. Subjects could examine items of information about each charity before making their choice. The data consist of a sequence of durations corresponding to the amounts of time a subject spent examining the information items before coming to a decision. The number of items the *j*th subject inspected (*N_j_*) is a random variable, and so is the amount of time spent on each item (*Z_ij_*).

One of the experimental variables was the similarity of the charities to one another. Similarity had two levels, low and high. The psychological meaning of the amount of time spent examining a piece of information arguably is quite different from the meaning of the number of pieces of information examined by a decision maker, and one of the researcher's goals was to ascertain whether similarity among options, a characteristic known to make decisions more “difficult,” would differentially affect inspection time per item vs. number of items examined. He also wished to ascertain the joint effect on the total time to decision, especially in the event that the effects turned out to be in opposite directions (this last possibility has some basis in the decision literature, but we do not go into that here).

A compound Poisson-gamma GLM predicting *n_j_* from similarity yields α = −0.069 with a 95% credible interval (CI) [−0.330, 0.201]. A two-level LN model predicting mean fixation duration from similarity yields β = 0.306 with a 95% CI [0.064, 0.550]. The high-similarity items take significantly longer for the subjects to process. Does this effect translate into significantly longer total time to make a decision in the high-similarity condition?

Using our heuristic sum of the coefficients to predict the effect size, the coefficient for the log mean duration is 0.306 and the coefficient for the log frequency is −0.069, so their combined effect should yield a coefficient of approximately 0.237 if we model the duration sums using the log link. A Bayesian hierarchical LN model (predicting the sum as a log-normal variate) yields a coefficient estimate of the similarity effect equaling 0.237, our predicted value, with 95% CI [−0.118, 0.596]. This time, the combination of a non-significant frequency effect and significant mean duration effect has not produced a significant effect on the duration sum. Instead, the additional noise introduced by random variation of the frequencies has “washed out” the effect on the means. As in the Owen et al. eye-tracker study, this example also underscores the importance of modeling the sums of the *Z_ij_* rather than attempting to intuitively infer effects on the sums from effects on the frequencies and means.

### 2.4. Model evaluation and diagnostics

The usual model diagnostics are available for the frequency and magnitude models, and we will not dwell on those here. Instead, we focus on diagnostics for the sums model. Both the simulation-based and Bayesian models enable the modeler to compare the predictive sums distribution against the observed sums distribution. Both approaches also share the capacity to informally evaluate goodness-of-fit for the fixed-effects component of the sums model, via conventional summary statistics such as the correlation between fixed-effects predictions and the observed sums.

**Figure 1:**
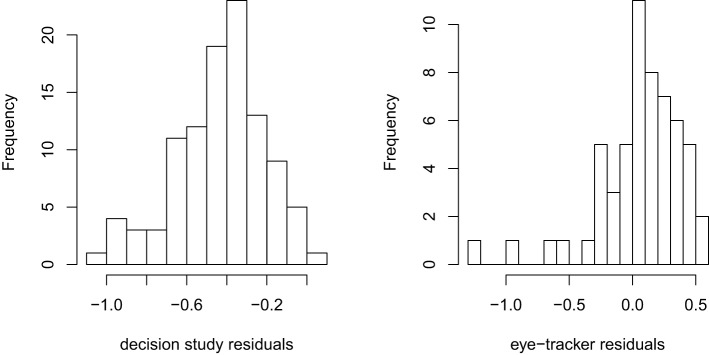
**Residuals histograms**.

The Bayesian approach, however, has diagnostic capabilities well beyond those of the simulation-based approach. We can recover the predicted sums for every case, incorporating the random intercepts, thereby enabling diagnostics via residuals, leverage, and influence statistics. Figure 1 shows histograms of the residuals from the decision task and eye-tracker studies sums LN models, in the log-scale. The decision study histogram reveals a reasonably normal-looking distribution exhibiting the aforementioned downward bias. However, the eye-tracker residuals have a few definite outliers, suggesting that these might be influential cases. Cross-validation and other resampling methods also may be applied here, although they are computationally intensive.

Formal model comparison is not straightforward because the usual model comparison methods, such as likelihood-ratio tests, are not available. However, informal comparisons via goodness-of-fit statistics may be employed. In the Bayesian approach the posterior mean of the deviance and Deviance Information Criterion (DIC, Spiegelhalter et al., 2002) are available for the frequency, magnitude, and sums models simultaneously. In the eye-tracker example, a null LN model yields a posterior mean deviance of 1088.0 and DIC = 1091.0 for the sums, whereas when the effects are included in the model, the sums model's posterior mean deviance is 1086.0 and DIC = 1090.0, indicating a small improvement over the null model.

Likewise, different GLMs may be compared with each other for predictive accuracy and goodness of fit. However, these indicators may not always agree with one another. The correlations between the observed means and sums and their posterior estimates for the GG model are 0.903 and 0.978, respectively, while for the LN model the correlations are 0.991 and 0.816. The DIC values reverse the order of performance for the sums but echo it for the magnitude means. For the GG model the DIC values are 1106 for the sums and 29040 for the magnitudes, whereas for the LN model are 1090 for the sums (better than the GG model) and 28560 for the magnitudes (also better).

Finally, model diagnostics may provide a basis for deciding between disagreeing models. In the decision task study, while the LN model estimates the effect of greater similarity on magnitudes as 0.306 with a 95% CI of [0.064, 0.550], the GG model's estimate is 0.179 with a 95% CI of [−0.042, 0.399]. The correlations between the observed means and sums and their posterior estimates for the GG model are 0.996 and 0.999, respectively, while for the LN model the correlations are 0.927 and 0.962, suggesting that the GG model is superior in both estimations. This time the DIC values reverse the order of performance for the means but echo it for the sums. For the GG model the DIC values are 2574 for the sums and 99960 for the magnitudes, whereas for the LN model are 2813 for the sums (worse, in agreement with the correlations) but 99810 for the magnitudes (better). The balance of evidence seems to favor the GG model. The evaluation of competing GLMs for randomly stopped sums on the basis of indicators, such as these, remains an active area of research.

## 3. Model bias, Type I error, and power

The models for the sums are approximations that may not be accurate for small sample sizes, and likewise the relationship between their power and that of the frequency and magnitude models is unknown. We conducted simulations to investigate the accuracy of the Type I error-rates when the null hypothesis is true, and power when it is false. The simulations required appropriate distributions for magnitudes and frequencies. For magnitudes, in the no-effects condition we used log-normal and gamma distributions with means of approximately 1000 and standard deviations of about 500. Values such as these are common in studies of human response times (measured in milliseconds) for a simple task such as a button-press. Given this mean and standard deviation, the log-normal distribution had a mean in the log scale of 6.81 and standard deviation of 0.447, while the gamma distribution had a shape parameter value 4 and scale parameter value 250.

For frequencies, we used a negative binomial distribution because overdispersion is commonly observed in counted data. The choice of parameter values was again based on experience with research where our technique is likely to find application. The negative binomial distribution can be parameterized in terms of an event probability, π, and scale parameter, ϕ. The expected frequency is θ = ϕπ/(1 − π) and the variance is ω^2^ = ϕπ/(1 − π)^2^. For the no-effects condition we chose π = 0.5 and ϕ = 10, resulting in an expected frequency 10 and variance 20. The simulations were run with two models for magnitudes and sums: The log-normal and gamma models.

To simulate effects, we employed a simple two-condition design with equal sample sizes of 25, 50, 100, and 200 in each condition. Effect sizes for both frequency and magnitude were in standard deviation units: ±0.2, ±0.5, and ±0.8. These correspond to Cohen's (1988: 25–27) “small,” “medium,” and “large” effect sizes. The positive and negative effects are needed because of the asymmetric distributions. We expected there to be greater power to detect decreases in expected values (negative effects) than equivalent increases (positive effects). There were four effects scenarios: A magnitude effect only, a frequency effect only, equal magnitude and frequency effects in opposing directions, and equal magnitude and frequency effects in the same direction. Thus, for each magnitude distribution there were 24 simulations for the first scenario, 24 for the second, 48 for the third, and 24 for the fourth. Each simulation had 10,000 runs and the simulations were coded in R version 2.15 (2013).

The effects from the magnitude predictors are accurately estimated in both the magnitude and sums models. The effects from the frequency predictors tend to be slightly over-estimated in the frequency model, and there is also upward bias in the sums model estimates when a predictor contributes to the frequency model. Table 2 shows the Type I error-rates from the log-normal and gamma distribution simulations. There is a clear but mild Type I error-rate inflation in all three models (magnitude, frequency, and sums) that tends to decrease with larger samples. This inflation is slightly greater for the sums models when the magnitudes are gamma-distributed, regardless of whether the model itself assumes a log-normal or gamma distribution.

**Table 2:** **Type I error rates**.

***N***	**Log-normal model**			**Gamma model**		
	**Magnitude**	**Frequency**	**Sums**	**Magnitude**	**Frequency**	**Sums**
**LOG-NORMAL DISTRIBUTION**						
25	0.0570	0.0572	0.0565	0.0549	0.0565	0.0570
50	0.0512	0.0531	0.0529	0.0537	0.0532	0.0524
100	0.0513	0.0513	0.0531	0.0544	0.0518	0.0523
200	0.0529	0.0514	0.0500	0.0517	0.0508	0.0497
**GAMMA DISTRIBUTION**						
25	0.0567	0.0572	0.0647	0.0553	0.0580	0.0652
50	0.0520	0.0521	0.0612	0.0538	0.0528	0.0606
100	0.0502	0.0531	0.0618	0.0511	0.0520	0.0611
200	0.0515	0.0514	0.0609	0.0488	0.0528	0.0593

Our investigations into power begin with the cases where there is only an effect on magnitudes vs. cases where there is only an effect on frequencies. Tables 3, 4 display the results of simulations for both kinds of cases, for the log-normal model only (the gamma model produced very similar results). The power to detect the relevant effect is located to the left of the resultant power for the sums model. For example, in both tables, as expected, it is evident that the sums model has greater power to detect negative than equivalent positive effects. This is due to the positive skew in the magnitude and frequency distributions and their lower-bounded supports, whereby a shift downwards changes a greater proportion of the distribution than an equivalent shift upward. This difference in power is most pronounced for small to moderate samples and effects.

**Table 3:** **Power for magnitude effects only**.

***N***	**Mag. = 0.2**	**Sum**	**Mag. = 0.5**	**Sum**	**Mag. = 0.8**	**Sum**
25	0.4734	0.0880	0.9921	0.2800	1.0000	0.5530
50	0.7461	0.1278	1.0000	0.4933	1.0000	0.8550
100	0.9653	0.2266	1.0000	0.7918	1.0000	0.9863
200	0.9997	0.3800	1.0000	0.9745	1.0000	1.0000
***N***	**Mag. = −0.2**	**Sum**	**Mag.= −0.5**	**Sum**	**Mag. = −0.8**	**Sum**
25	0.5608	0.0920	0.9997	0.4183	1.0000	0.8730
50	0.8369	0.1512	1.0000	0.7052	1.0000	0.9914
100	0.9863	0.2579	1.0000	0.9366	1.0000	1.0000
200	1.0000	0.4384	1.0000	0.9987	1.0000	1.0000

**Table 4:** **Power for frequency effects only**.

***N***	**Freq. = 0.2**	**Sum**	**Freq. = 0.5**	**Sum**	**Freq. = 0.8**	**Sum**
25	0.0957	0.0900	0.3682	0.3189	0.6779	0.6017
50	0.1484	0.1292	0.6174	0.5568	0.9321	0.8958
100	0.2644	0.2362	0.9027	0.8602	0.9988	0.9963
200	0.4880	0.4311	0.9964	0.9906	1.0000	1.0000
***N***	**Freq. = −0.2**	**Sum**	**Freq. = −0.5**	**Sum**	**Freq. = −0.8**	**Sum**
25	0.1131	0.1098	0.5240	0.4765	0.9195	0.8758
50	0.1882	0.1703	0.7908	0.7429	0.9949	0.9890
100	0.3220	0.2851	0.9719	0.9523	1.0000	1.0000
200	0.5688	0.5135	0.9998	0.9992	1.0000	1.0000

Inspection of both tables reveals that the power of the sums model is considerably less than the power of the magnitude model but nearly identical to power of the frequency model. The explanation for this inheres in the observation that in a two-level data structure, the variation in the sums is at the same top level as the variation in frequencies, whereas the variation in the magnitudes is at the bottom level. Thus, the variance of the sums will correspond more closely to the variance of the frequencies. However, this does not mean that the power of the sums model is not influenced by the power of the magnitude model.

Results thus far suggest that frequency effects exert greater influence on the sums model than equivalently large magnitude effects. This suggestion is borne out by simulations in which equal-sized but opposite-signed frequency and magnitude effects occur simultaneously. Simulations under this condition revealed a somewhat counter-intuitive finding here is that if the frequency and magnitude effects have opposite signs but equal sizes then the net outcome for the sums model is a negative effect. Table 5 shows that power is greater when the negative effect is produced by the frequencies than when it is produced by the magnitudes. The overall net negative effect finding is due to the greater power to detect negative effects as determined by the asymmetric frequency and magnitude distributions, so that the sums models tend to yield negative effects no matter whether magnitude effects or frequency effects are taking the negative role. The results in Table 5 are for the log-normal model, but the gamma model again produced very similar results.

**Table 5:** **Power for frequency and magnitude effects in opposite directions**.

	**Mag. = 0.2**	**Mag. = 0.5**	**Mag. = 0.8**
***N***	**Freq. = −0.2**	**Freq. = −0.5**	**Freq. = −0.8**
25	0.0391	0.0924	0.2343
50	0.0408	0.1223	0.3793
100	0.0420	0.1833	0.6197
200	0.0524	0.3087	0.8832
	**Mag. = −0.2**	**Mag. = −0.5**	**Mag. = −0.8**
***N***	**Freq. = 0.2**	**Freq. = 0.5**	**Freq. = 0.8**
25	0.0243	0.0425	0.1177
50	0.0232	0.0501	0.2154
100	0.0238	0.0719	0.3855
200	0.0256	0.1035	0.7050

In the eye-tracking example we found that a non-significant frequency effect and non-significant magnitude effect could combine to yield a significant effect on sums. This finding suggests that there are conditions under which the power to detect an effect may be greater in the sums model than in its “constituent” frequency and magnitude models. To investigate this possibility, we conducted simulations with sample sizes of 25 and 50 in each condition for a two-condition design, with same-signed combinations of effect-sizes of ±0.2 and ±0.5 standard deviation units. Table 6 displays the results. In each subtable, the only cell in which the power for the sums model exceeds the power of both the frequency and magnitude models is where the frequency effect size is ±0.5 and the magnitude effect size is ±0.2. We conclude that power in the sums model can be greater than power to detect magnitude effects and frequency effects, when magnitude and frequency effects are in the same direction and when the frequency effect is larger than the magnitude effect.

**Table 6:** **Power for frequency and magnitude effects in the same direction**.

***N* = 25**	**Magn**.	**Effect**	
**Freq. effect**	**0.2**	**0.5**	**Freq. power**
0.2	0.2334	0.5180	0.1011
0.5	0.5252	0.8191	0.3623
**Magn. power**	0.4983	0.9948	
**Freq. effect**	**−0.2**	**−0.5**	**Freq. power**
−0.2	0.2653	0.6801	0.1204
−0.5	0.6791	0.9347	0.5095
**Magn. power**	0.5653	0.9996	
***N* = 50**	**Magn**.	**Effect**	
**Freq. effect**	**0.2**	**0.5**	**Freq. power**
0.2	0.3834	0.8125	0.1455
0.5	0.8369	0.9848	0.6233
**Magn. power**	0.7949	1.0000	
**Freq. effect**	**−0.2**	**−0.5**	**Freq. power**
−0.2	0.4859	0.9283	0.1909
−0.5	0.9338	1.0000	0.7923
**Magn. power**	0.8514	1.0000	

## 4. Extensions

We now investigate two extensions to the randomly stopped sums model. The first of these is going beyond a two-level model to incorporate three or more levels. Although this could be achieved with the simulation approach, the hierarchical Bayesian method is simpler and more principled, so that is the approach elaborated in the following subsection. The second extension is the incorporation of observation-level predictors into the sums model. Examples of applications for this extension include consumer ratings of their desire for a product at the time they purchase it, or a decision maker's rating of the difficulty of each sub-task leading to their decision.

### 4.1. Multi-level models via a Bayesian approach

As a demonstration of the generalizability and flexibility of the hierarchical Bayesian approach to modeling randomly stopped sums, we present our reanalysis of data from another study by Tang (2013). In an online experiment, 134 participants were asked to choose which of two decks of reward or loss cards gave them greater rewards or lesser penalties. They were allowed to sample a randomly chosen card from each of the decks as many times as they wished before coming to a decision. As in the preceding decision task example, the dependent variables in this study were the number of times the participant inspected either deck, the amount of time spent on each inspection, and the total amount of time taken to reach a decision. Each participant completed four rounds (i.e., four decision tasks of this kind). The rounds were distinguished by whether the decks' means differed or the variances differed, and whether the cards gave rewards or penalties. These two factors were counterbalanced for each participant. The relevant hypotheses were that participants would take longer and/or require more inspections to decide when the decks penalized, and likewise when the decks' variances differed but the means were identical.

Participants were randomly assigned to one of three experimental conditions: A “control” condition where they were given a distractor task at the outset (rearranging jumbled sentences), a “strategic prime” condition in which they were advised beforehand to carefully consider how best to get the most rewards from the decks, and a “self-reflective” condition in which they were advised beforehand to focus on their thinking, feelings, and decision making processes. The hypothesis here was that the strategic prime and the self-reflective conditions would induce longer inspection times and/or a greater number of inspections.

Finally, two covariates were measured as potential influences on decisiveness. One covariate measures the extent to which a person prefers to avoid decisions and the other measures the degree to which a person finds decision making aversive. The motivation for including these covariates stems from the notion that indecisiveness can arise either because a person avoids or delays decision making, or because they become ensnared in obsessing over details in the decision making process. The relevant hypotheses were that avoidance would not predict time taken or number of inspections required to make a decision, whereas aversion would predict these.

Because the experimental design includes a random number of inspections for each participant within each of four rounds, the data structure for inspection times has three levels: Inspections, rounds, and participants. Likewise, the number of inspections has two levels: Rounds and participants.

The three-level hierarchical model is a straightforward generalization of the two-level model described in the section on Bayesian methods. The frequencies have two levels, so the Poisson distribution parameter is modeled by a gamma distribution whose parameters accommodate this, i.e., λ_*jk*_ ~ Gamma(*q*, *r_jk_*). The *q* parameter, as before, is given an uninformative gamma prior. The *r_jk_* = *q*/exp(η_*jk*_). The η_*jk*_ are modeled by

(11) $$\eta_{jk}=\alpha_{0}+\alpha_{1}d_{1j}+\alpha_{2}d_{2j}+\alpha_{3}x_{1j}+\alpha_{4}x_{2j}+\alpha_{5}f_{k}+\alpha_{6}t_{k},$$

where *k* indexes the four rounds, *d*_1*j*_ and *d*_2*j*_ are binary indicator variables for the strategic and self-relfective conditions, *x*_1*j*_ is the aversion score, *x*_2*j*_ is the avoidance score, *f_k_* is a binary indicator for the gain frame, and *t_k_* is a binary indicator for the type where the decks' means differ. As before, the α_*m*_ coefficients are given uninformative Gaussian priors.

For the LN model, the magnitudes *Z_ijk_* are modeled as log-normal variates with means μ_*jk*_ in the log-scale and variance σ^2^. The μ_*jk*_ ~ *N*(υ_*jk*_, σ^2^_υ_), with the υ_*jk*_ modeled by

(12) $$\upsilon_{jk}=\beta_{0k}+\beta_{1}d_{1j}+\beta_{2}d_{2j}+\beta_{3}x_{1j}+\beta_{4}x_{2j}+\beta_{5}f_{k}+\beta_{6}t_{k}.$$

Note that here we have separate intercepts, β_0*k*_, for the four rounds. Likewise, the β*_m_* coefficients are given uninformative Gaussian priors. Finally, the sums are modeled as log-normal variates having means μ_Sjk_ in the log-scale and variance σ^2^*_s_*, with

(13) $$\begin{array}{l} \mu_{\text{S}jk}=\omega_{0k}+\omega_{1}d_{1j}+\omega_{2}d_{2j}+\omega_{3}x_{1j}\\ \;+\omega_{4}x_{2j}+\omega_{5}f_{k}+\omega_{6}t_{k}. \end{array}$$

For the GG model, the magnitudes are modeled as gamma(*q_m_*, *p_jk_*) variates, with *p_jk_* = *q_m_*/exp(μ_*jk*_), μ_*jk*_ defined as above. The sums, then, are modeled as gamma(λ*_jk_q_m_*, *p_jk_*) variates.

The resultant models converge well in all parameters and require a 5,000 iteration burn-in to do so. The LN model is outperformed by the GG model, on grounds of both better DIC values and stronger correlations between predicted and observed data. The GG model reproduces the duration means, frequencies, and sums very well, with multiple *R*^2^ values of 0.9997, 0.9999, and 0.9999, respectively. We therefore discuss only the GG model estimates here.

Table 7 presents the model coefficients for the frequency and magnitude models, and Table 8 displays the coefficients for the sums model. In Table 7 we can see that the only clear effect on frequencies is from the types variable (α_6_ = −0.093, so that the different-means comparison takes less time than the different-variances comparison).

**Table 7:** **Three-level GG model frequency and magnitude coefficients**.

			**95% credible interval**	
	**Param. Estim**.	***SE***	**Lower**	**Upper**
α_0_	1.245	0.021	1.203	1.287
α_1_	−0.030	0.028	−0.083	0.024
α_2_	0.003	0.026	−0.050	0.053
α_3_	−0.010	0.011	−0.032	0.011
α_4_	−0.017	0.011	−0.039	0.005
α_5_	−0.005	0.011	−0.026	0.015
α_6_	−0.093	0.022	−0.137	−0.049
β_01_	6.098	0.055	5.991	6.206
β_02_	5.988	0.054	5.882	6.095
β_03_	5.934	0.055	5.825	6.040
β_04_	5.898	0.053	5.793	6.002
β_1_	0.152	0.052	0.051	0.252
β_2_	0.121	0.049	0.025	0.218
β_3_	0.016	0.021	−0.025	0.057
β_4_	0.131	0.021	0.089	0.172
β_5_	0.006	0.020	−0.033	0.046
β_6_	0.013	0.040	−0.066	0.090

**Table 8:** **Three-level GG model sums coefficients**.

			**95% credible interval**	
	**Param. Estim**.	***SE***	**Lower**	**Upper**
ω_01_	7.343	0.059	7.229	7.458
ω_02_	7.233	0.059	7.119	7.346
ω_03_	7.179	0.059	7.062	7.294
ω_04_	7.143	0.057	7.029	7.254
ω_1_	0.122	0.058	0.009	0.238
ω_2_	0.124	0.055	0.014	0.232
ω_3_	0.006	0.024	−0.041	0.052
ω_4_	0.113	0.024	0.067	0.160
ω_5_	0.001	0.023	−0.044	0.047
ω_6_	−0.080	0.046	−0.170	0.010

There are three effects on the durations (in this example, magnitudes are durations). As expected, participants in the strategic and self-reflective conditions take longer on average for an inspection than participants in the control condition. Likewise, participants with higher avoidance scores take longer for an average inspection. However, aversion scores do not appear to yield an effect, nor does frame or type.

In Table 8 it is clear that all three of the effects on duration have translated into effects for the sums of the durations, despite the absence of corresponding effects on frequencies. Thus, participants in the strategic and self-reflective conditions and participants with higher avoidance scores take longer for overall than their opposite numbers. However, the effect of type on frequencies has not yielded an effect on duration sums.

**Figure 2:**
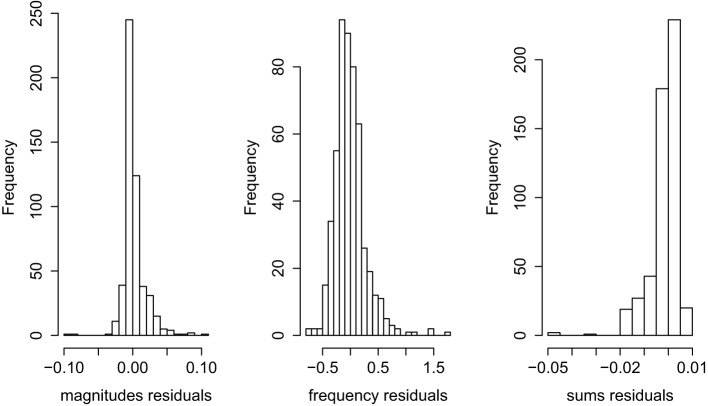
**Residuals for three-level GG model**.

Turning now to model diagnostics, the residuals plots in Figure 2 reveal a moderate skew in the sums residuals and a few outliers in both the magnitudes (means) and sums residuals. However, given the large number of observations in the dataset (the average number of items inspected in each round was 28.5, and so 134 participants with 4 rounds result in 15,276 observations), these outliers have no discernible impact on the model coefficients or standard errors.

### 4.2. Observation-level predictors

We now propose an extension of the sums model to incorporate observation-level predictors of the magnitudes *Z_ij_*. Equation (3) for the magnitude model is modified to do this as follows:

(14) $$g\left(Z_{ij}\right)=\mu_{j}+\tau_{ij}+u_{ij},$$

where

(15) $$\tau_{ij}=\sum_{p}\delta_{pj}w_{pij},$$

*u_ij_* ~ *N*(0, σ_*u*_), and random coefficients δ_*pj*_ = η*_p_* + ϵ_*pj*_ with ϵ_*pj*_ ~ *N*(0, σ_*p*_), and the *w_p_* are the predictors.

This model component may be included in the sums model by observing that its cumulative effect for the *j*th subject is just the sum of the τ_*ij*_ over *i*, which entails extending Equation (6) in the following way:

(16) $$\mu_{Sj}=\sum_{k}\alpha_{k}x_{kj}+\sum_{m}\beta_{m}y_{mj}+\sum_{p}\eta_{p}w_{p.j}.$$

## 5. Conclusion

Modeling randomly stopped sums with a life distribution with a log link function enables modelers to explore how the effects from experimental variables or covariates on frequency and magnitudes combine into effects on the sums. The Bayesian approach enriches the capacity of the sums models by integrating the frequency, magnitude, and sums models into a single hierarchical model, and enlarging the range of model diagnostic and evaluative tools. These two innovations overcome in good part what could be regarded by researchers in psychology as problematic limitations in the standard financial statistical versions of these models. The model developed in this paper is fairly general, as it can incorporate various discrete distributions for the frequencies and continuous distribution for magnitudes, so long as a log link function is used for each of them. It is also noteworthy that both the frequency and magnitude models can incorporate autocorrelation terms, the λ_*j*_ model can incorporate over-dispersion, the μ_*j*_ can incorporate random slopes, and the ε*_j_* variance can have its own submodel to deal with heteroscedasticity.

The simulation studies summarized in this paper indicate that the Type I error rates may be slightly liberal for small samples and effect sizes, but this should not be problematic for most practical purposes. The simulations confirmed the intuitions that the power of the sums model is more directly influenced by the power of the frequency model than the power of the magnitude model, and it is easier to resolve negative effects than positive effects of the same size. Nevertheless, perhaps the key finding regarding power is that, for two-level data structures, the sums model's power can exceed that of the frequency and magnitude models when they share effects in the same direction and the frequency model effect is greater than the magnitude model effect. This finding also underscores the utility of modeling the sums in addition to modeling the frequencies and magnitudes.

This type of model should find wide application in several areas in psychology, notably those in which a psychological process is thought to be serially summed from observable component process outputs, when the output of the component processes is a non-negative random variable and the number of such components also is a random variable. We have given examples from perception and decision making. Models for randomly stopped sums will be most useful when the sums are operationalizations of psychologically meaningful constructs that are considered distinct from the magnitudes and frequencies. The eye-tracker studies are an example, because the total amount of visual attention given to an advertisement or part of a webpage is the chief interest there, instead of the number of fixations or average fixation duration. Another example is cumulative hours of deliberative practice as a proxy indicator of expertise. Neither the average duration of practice sessions nor practice session frequency measures accumulated expertise, whereas the sum of the durations does so. Other topics that come readily to mind include purchasing behavior, food or drug consumption, cumulative exposure to risks, cumulative stress, and generally cumulative expenditure of time on any episodic activity (e.g., dwell time on webpages in a web-browsing session).

### Conflict of interest statement

The authors declare that the research was conducted in the absence of any commercial or financial relationships that could be construed as a potential conflict of interest.
